# The association between fat mass and obesity‐associated (*FTO*) genotype and serum vitamin D level in breast cancer patients

**DOI:** 10.1111/jcmm.16908

**Published:** 2021-09-06

**Authors:** Maryam Gholamalizadeh, Zohreh Mokhtari, Saeid Doaei, Vahideh Jalili, Sayed Hossein Davoodi, Mona Jonoush, Mohammad Esmail Akbari, Azadeh Hajipour, Bojlul Bahar, Ghasem Azizi Tabesh, Saeed Omidi, Seyed Alireza Mosavi Jarrahi

**Affiliations:** ^1^ Student Research Committee Cancer Research Center, Shahid Beheshti University of Medical Sciences Tehran Iran; ^2^ Department of Clinical Biochemistry Faculty of Medicine Shahid Beheshti University of Medical Sciences Tehran Iran; ^3^ Research Center of Health and Environment Guilan University of Medical Sciences Rasht Iran; ^4^ Faculty of Medicine Urmia University of Medical sciences Urmia Iran; ^5^ Departments of Clinical Nutrition and Dietetics Faculty of Nutrition and Food Technology National Nutrition and Food Technology Research Institute Shahid Beheshti University of Medical Sciences Tehran Iran; ^6^ Department of Nutrition School of Medicine Mashahd University of Medical Sciences Mashahad Iran; ^7^ Cancer Research Center Shahid Beheshti University of Medical Sciences Tehran Iran; ^8^ School of Health Qazvin University of Medical Sciences Qazvin Iran; ^9^ Nutrition Sciences and Applied Food Safety Studies Research Centre for Global Development School of Sport & Health Sciences University of Central Lancashire Preston UK; ^10^ Department of Medical Genetics School of Medicine Shahid Beheshti University of Medical Sciences Tehran Iran; ^11^ Department of Health Education and Promotion Research Center of Health and Environment School of Health Guilan University of Medical Sciences Rasht Iran; ^12^ Cancer Research Center Shahid Beheshti University of Medical Sciences Tehran Iran

**Keywords:** breast cancer, *FTO* gene, polymorphism, vitamin D

## Abstract

The preventive effect of vitamin D against breast cancer can be influenced by gene polymorphisms. This study aimed to investigate the association between serum level of 25(OH) vitamin D and *FTO* genotype in breast cancer patients. A cross‐sectional study was carried out on 180 newly diagnosed patients with breast cancer in Tehran, Iran. The blood samples were collected from the participants in order to assess the *FTO* gene rs9939609 polymorphism by the tetra‐primer amplification refractory mutation system (Tetra‐ARMS) PCR method. The serum level of 25(OH) vitamin D was measured using the direct competitive enzyme‐linked immunosorbent assay (ELISA) method. The association between vitamin D and the *FTO* genotype in patients with breast cancer was assessed after adjustment for cofounders. The frequency of TT, AT and AA genotypes in the breast cancer patients were 43% (*n* = 77), 49% (*n* = 89) and 8% (*n* = 14), respectively. All patients with higher than 40 ng/dl of serum 25(OH) vitamin D had one or two copies of *FTO* rs9939609 risk allele (*p* = 0.019). No linear association was found between the number of FTO risk allele and the level of serum vitamin D. All patients with high serum level of 25(OH) vitamin D had one or two copies of *FTO* rs9939609 risk allele. *FTO* gene polymorphisms may counteract the beneficial effects of vitamin D in breast cancer prevention. Further studies can help to better understand the genetic factors predisposing to breast cancer and their effect on the association between vitamin D and breast cancer.

## 1 INTRODUCTION

Breast cancer (BC) is a common malignant tumour in women, accounts for 10% of malignant tumours and the second prevalent after uterine endometrial carcinoma.^1^, ^2^ BC is the leading cause of cancer deaths in women, and there are approximately one million new cases diagnosed in the world every year.^3^ A family history of BC is strongly associated with a significant increase in the risk of BC. However, familial BCs linked to high penetrant genetic mutations account for only about 5% of cases.^4^ Recent studies reported that obesity and excess calorie intake can increase BC risk^5^, ^6^, and there is growing evidence that weight loss and decrease in fat consumption may lead to a decrease in the risk for developing BC.^7^, ^8^

The fat mass and obesity‐associated (*FTO*) gene is frequently reported to be associated with obesity and some recent studies reported that dysregulation of *FTO* gene expression can affect multiple cancer‐related processes, such as cancer cell apoptosis, proliferation, migration, invasion, metastasis, cell‐cycle and differentiation in BC patients.^9^ A significant association between *FTO* rs9939609 polymorphism risk allele (A) and BC risk was identified in females with overweight.^10^ This association mostly relied on the role of *FTO* in specific signalling pathways including the phosphoinositide 3‐kinase/protein kinase B/ mechanistic target of rapamycin **(**PI3K/AKT/ mTOR) and mitogen‐activated protein kinase (MAPK) signalling pathways that are associated with cell growth, cell proliferation and inflammatory immune responses.^9^, ^11^

On the other hand, some vitamins may have a role in the prevention of BC. Vitamin D and its receptor (VDR) can regulate the expression of cancer‐related genes in a wide range of mammalian cell types.^12^, ^13^ Growing evidence suggest that lower levels of vitamin D may contribute to an increased risk of BC.^14^ Recent studies reported that gene polymorphisms may regulate the association between serum 25(OH)D and BC.^15^ For this, polymorphisms present in the *FTO* gene were investigated and the interactions between *FTO* genotype, BC and vitamin D serum level were reported.^12^, ^13^, ^14^, ^15^ A recent study reported that the effect of rs9939609 risk allele on body weight may be limited to people with insufficient vitamin D intake.^10^ However, the association of *FTO* genotype and serum level of vitamin D in BC patients is not yet clear. Hence, this study aimed to investigate the association of serum level of vitamin D with *FTO* rs9939609 polymorphism in Iranian BC patients.

## 2 METHODS

### 2.1 Study population and data collection

This cross‐sectional study included 180 newly diagnosed patients with BC and performed from January 2019 to August 2020. The sample size was calculated using Open EPI software and similar previous studies.^16^, ^17^ The participants were selected according to the inclusion criteria from the female patients referring to the cancer research centre of Shohadaye Tajrish Hospital in Tehran, Iran. The inclusion criteria included females with confirmed BC, diagnosed recently, age between 35 and 70 years and consent to participate in the study. Basic information including medical history, alcohol consumption, smoking, the level of education and socioeconomic factors was collected. The amount of calorie and vitamin D intake in the past year was assessed using a validated Food Frequency Questionnaire (FFQ).^18^ The level of physical activity was measured using the International Physical Activity Questionnaire (IPAQ) which was validated in a previous study.^19^ The patient's weight and height were measured using a SECA scale (Alpha 882, SECA Corporation, Hamburg, Germany), and their height was measured using a stadiometer, respectively. The BMI was calculated by dividing the weight (kg) by the square of height (m^2^).

### 2.2 Genotyping

At the beginning of the study, from each participant, about 5 ml intravenous blood sample was collected onto an ethylenediaminetetraacetic acid (EDTA) tube (Becton Dickinson, France) by a trained phlebotomist. The blood sample was stored at −70 C until used for further analysis. The genomic deoxyribonucleic acid (DNA) was extracted from the blood samples using GeneAll DNA extraction kit (Incheon, Korea) following the manufacturer's instruction. Polymerase chain reaction (PCR) was performed with 50 ng of genomic DNA using a PCR amplification instrument (GeneQ; Hangzhou Bioer Technology Co., Ltd.) and master mix DNA polymerase (cat. No A180301; Ampliqon). The PCR products were examined to identify rs9939609 polymorphism of the *FTO* gene by the tetra‐primer amplification refractory mutation system (Tetra‐ARMS) PCR method.

### 2.3 Determination of serum level of vitamin D

From an aliquot (3 ml) of frozen blood sample, the serum was prepared by centrifugation at 1500 g for 15 min using a Universal centrifuge (Wehingen). Quantification of serum 25‐hydroxy vitamin D was performed using the vitamin D VIDAS Kit (Marcy‐l'Étoile, bioMérieux, France). This kit is based on a direct competitive enzyme‐linked immunosorbent assay (ELISA) based method. The VIDAS 25‐OH vitamin D total assay is considered as a suitable measuring method for vitamins D2 and D3 serum levels with a high accuracy. Correlation between the results from VIDAS kit with the reference methods of chromatography and volume spectrometry was r=0.93 which indicated the high efficiency of this method. The levels of higher than 40 ng/dl of serum vitamin D were considered as high level, based on the previous studies on cancer‐protective effects of vitamin D.^15^

### 2.4 Statistical analysis

The patients with different *FTO* genotypes and different level of serum vitamin D were compared in terms of demographic, anthropometric and clinical indices using t test (for quantitative variables) and chi‐square (for qualitative variables) methods. Then, the association between the frequency of the risk allele A of rs9939609 polymorphism and serum levels of vitamin D was investigated using the linear regression method and the effects of confounding variables including age, Stage, Pregnancy, Marriage, Family history of BC, Menopause and Abortion (model 2) and BMI, smoking, alcohol, physical activity and dietary intake of calorie, protein, carbohydrate and vitamin D (model 3) were adjusted. All statistical analyses were performed using SPSS software ver. 21.0 (IBM), and the significance level of *p* < 0.05 was considered for all analyses.

### 2.5 Ethics approval and consent to participate

All patients signed an informed consent form at baseline. This study was approved by the ethical committee of Shahid‐Beheshti University of Medical Sciences (code: IR.SBMU.RETECH.REC.1398.784).

## 3 RESULTS

The frequency of TT, AT and AA genotypes were 43% (*n* = 77), 49% (*n* = 89) and 8% (*n* = 14), respectively. The distribution of demographic, anthropometric, physiological and pathological characteristics in the BC patients based on different *FTO* genotypes (TT, AA and AT) for the rs9939609 and different serum levels of vitamin D are presented in Table 1. The mean age of TT carriers and AT/AA carriers were 59.57 (±11.66) and 57.73 (±11.11), respectively (***p*** > 0.05). No significant difference was found in terms of the anthropometry (height, weight and BMI), breastfeeding, menopause age, sleep duration stage of cancer, pregnancy, marital status, family history of BC, menopause and number of abortions between the patients with TT and AA/AT genotypes. However, when serum vitamin D levels were assessed, significant differences were evident when the patients were characterized based on the pregnancy (*p* < 0.05) and abortion history (*p* < 0.01) (Table 1).

**TABLE 1:** Distribution of demographic, anthropometric, physiological and pathological characteristics of the breast cancer patients along with their FTO genotype (rs9939609) and vitamin D level in the blood serum

Characteristics	FTO genotype (rs9939609)		*p*‐Value	Serum vit D level		*p*‐Value
	TT (*n* = 77)	AT/AA (*n* = 103)		Low vitamin D (*n* = 140)	High vitamin D (*n* = 40)	
Anthropometry						
Age (years)	59.57 ± 11.66	57.73 ± 11.11	0.520	57.89 ± 11.30	65.31 ± 10.49	0.920
Height (cm)	159.33 ± 5.09	160.86 ± 7.25	0.480	160.91 ± 7.21	160.42 ± 3.16	0.080
Weight (kg)	64.77 ± 9.23	71.45 ± 10.06	0.080	70.16 ± 9.79	73.71 ± 9.87	0.930
BMI (kg/m^2^)	25.48 ± 3.12	27.63 ± 4.46	0.120	27.13 ± 3.76	28.56 ± 3.04	0.260
Breastfeeding (month)	35.80 ± 31.53	38.92 ± 31.87	0.710	29.94 ± 28.17	37.53 ± 36.89	0.750
Menopause age (years)	54.00 ± 2.54	45.25 ± 4.64	0.190	49.20 ± 6.058	45.55 ± 5.40	0.850
Stage of cancer						
1	16 (21%)	24 (23%)	0.500	43 (31%)	18 (44%)	0.410
2	35 (46%)	48 (48%)		66 (47%)	12 (31%)	
3	22 (29%)	31 (29%)		29 (20%)	8 (19%)	
4	4 (4%)	0 (0%)		2 (2%)	2 (6%)	
Pregnancy (no.)						
<4	45 (58%)	57 (56%)	0.910	71 (51%)	13 (31%)	0.030
4– 8	31 (40%)	32 (31%)		67 (47%)	18 (44%)	
>8	1 (2%)	14 (13%)		2 (2%)	9 (25%)	
Marital status						
Single	3 (4%)	2 (2%)	0.440	6 (4%)	2 (5%)	0.400
Married	56 (72%)	82 (81%)		112 (80%)	37 (93%)	
Widowed	18 (24%)	18 (17%)		22 (16%)	1 (2%)	
Family history of breast cancer						
First degree	4 (5%)	7 (6%)	0.800	18 (13%)	14 (31%)	0.300
Second degree	5 (7%)	10 (9%)		31 (22%)	3 (6%)	
No	68 (88%)	86 (85%)		91 (65%)	23 (63%)	
Menopause						
Yes	75 (98%)	85 (84%)	0.320	123 (88%)	34 (86%)	0.740
No	2 (2%)	18 (16%)		7 (12%)	6 (14%)	
Abortion (number)						
<2	72 (94%)	91 (90%)	0.200	132 (94%)	35 (88%)	0.010
>2	4 (6%)	12 (10%)		8 (6%)	5 (12%)	
FTO genotype (rs9939609)						
TT				77 (54.9%)	0 (0%)	0.019
AA/AT				63 (45.1%)	40 (100%)	

*Categorized according to the mean (SD) or number (%).

The distribution of the status of lifestyle and dietary intake among different *FTO* genotypes and different serum levels of vitamin D are presented in Table 2. No significant difference was evident on smoking, alcohol consumption sleep duration, macronutrient/energy intake or blood serum level of vitamin D between TT and AA/AT genotypes. Based on the assessment of serum vitamin D levels and macronutrient intake characteristics, it was evident that the participants with a low level of vitamin D had a significantly (*p* < 0.01) higher daily intake of carbohydrates. All patients with higher than 40 ng/dl of serum 25(OH) vitamin D had one or two copies of *FTO* rs9939609 risk allele (*p* = 0.019) (Table 2).

**TABLE 2:** Lifestyle and the nutrient intake of the breast cancer patients with different FTO genotypes (rs9939609) and vitamin D levels in the blood serum

Parameters	FTO genotype (rs9939609)		*p*‐Value	Serum vitamin D level		*p*‐Value
	TT (*n* = 77)	AT/AA (*n* = 103)		Low vit D (*n* = 140)	High vit D (*n* = 40)	
Smoking						
Yes	1 (2%)	1 (1%)	0.410	4 (3%)	1 (2%)	0.830
No	99 (98%)	102 (99%)		136 (97%)	39 (98%)	
Alcohol consumption						
Yes	2 (3%)	1 (1%)	0.110	4 (3%)	1 (2%)	0.650
No	75 (97%)	102 (99%)		136 (97%)	39 (98%)	
Sleep duration (h/24 h)	7.30 ± 0.945	8.20 ± 2.15	0.780	7.73 ± 1.58	8.00 ± 1.15	0.410
Macronutrient/energy intake						
Energy (kcal/day)	2258.58 ± 121.65	2263.54 ± 1106.72	0.590	2315.52 ± 1141.52	2416.18 ± 588.85	0.070
Protein (g/day)	79.51 ± 44.73	85.21 ± 46.9	0.740	83.15 ± 35.10	84.38 ± 25.92	0.100
Carbohydrate (g/day)	336.93 ± 231.70	296.9 ± 149.29	0.640	318.58 ± 184.24	317.86 ± 174.21	0.006
Total fat (g/day)	95.66 ± 66.70	90.93 ± 56.70	0.850	93.15 ± 57.09	103.75 ± 53.56	0.690
Dietary vit D intake (mg/day)	1.35 ± 1.11	1.34 ± 1.22	0.970	1.7 ± 1.59	2.3 ± 1.23	0.360
Blood serum						
Vitamin D (ng/dl)	25.21 ± 8.86	29.06 ± 24.90	0.490	21.62 ± 9.42	58.66 ± 19.6	<0.001

No linear association was identified between the number of *FTO* risk allele and the level of serum vitamin D (Table 3). The results did not change after adjustment for age, breastfeeding, menopause age, sleep duration stage of cancer, pregnancy, marital status, family history of BC, menopause and number of abortions. The results remained nonsignificant after further adjustments for BMI, smoking, alcohol, sleep duration, physical activity and dietary intake of calorie, protein, carbohydrate and vitamin D.

**TABLE 3:** Linear regression of the association between FTO risk allele and serum vitamin D in patients with breast cancer

	*B*	*p*‐Value
Model 1	0.38	0.83
Model 2	0.21	0.43
Model 3	0.62	0.61

Notes

Model 1 crude. Model 2: Adjusted for age, stage of cancer, pregnancy, marriage, family history of breast cancer, Menopause, Abortion. Model 3. Further adjustments for BMI, smoking, alcohol, sleep duration, physical activity and dietary intake of calorie, protein, carbohydrate and vitamin D.

## 4 DISCUSSION

To our knowledge, this is the first study investigating the relationship of the *FTO* genotype and vitamin D in BC patients. The results indicated that the BC patients with risk allele (A) of *FTO* rs9939609 had significantly higher levels of vitamin D. Recent studies focused on the associations between BC with the *FTO* polymorphisms. It is frequently reported that obesity can increase the risk of BC in post‐menopause women,^20^, ^21^ and there is evidence that weight loss may reduce the risk of BC.^22^ However, Mojaver et al. found no significant association between the rs9939609 *FTO* gene and the risk of BC in Iranian women.^23^ Contradictory results were reported on the association between *FTO* gene and BC which can be due to the effects of different factors on the association between *FTO* gene and BC. For example, Kang et al. reported that BMI status could affect the association between rs9939609 polymorphism and BC and concluded that *FTO* gene polymorphism is probably associated with BC only in overweight individuals.^24^ Interestingly, the association between *FTO* gene polymorphism and BC has been reported to be influenced by the status of oestrogen receptors. Oestrogen may enhance BC cell proliferation by regulating *FTO* gene expression and activating the phosphatidylinositol 3‑kinase (PI3K)/protein kinase B (AKT) signalling pathway in oestrogen receptor‐positive patients. A systematic review examined the effect of diet on *FTO* gene expression in the hypothalamus and reported that macronutrient intake may regulate *FTO* gene expression.^25^ A recent case‐control study by Doaei et al., found no significant relationship between BC and the risk allele (A) of *FTO* rs9939609 in participants with normal BMI.^26^ in contrast with Zhao et al. indicating the association of *FTO* rs9939609 with BC risk.^27^ Mozaffarizadeh et al. reported that rs9939609 *FTO* gene polymorphism was significantly associated with BC risk in overweight individuals.^28^

On the other hand, an inverse relationship between vitamin D levels and BC risk has been reported in several studies. Extensive research has been done to identify the role of vitamin D in cancer prevention, and it was reported that vitamin D serum levels of above 40 ng/ml may be protective against BC.^15^ Mohr et al. in a meta‐analysis of 11 case‐control studies reported that individuals in the highest quintile of 25 (OH) vitamin D had a reduced risk of BC compared with the lowest quintile and the 25 (OH) vitamin D serum level of 47 ng/ml was associated with a 50% reduced risk of BC. A similar inverse relationship has been reported by Stoll et al. in a systematic review of 37 studies.^14^

Recent studies identified an association between serum vitamin D level and single nucleotide polymorphisms (SNPs) of some genes such as *FTO* gene. Vitamin D level was reported to be inversely related to obesity, and *FTO* gene may affect the association between vitamin D and obesity.^10^, ^29^ In the present study, the relationship between *FTO* genotype and 25 (OH) vitamin D was examined and all patients with more than 40 ng/dl serum 25 (OH) vitamin D (n = 40) had one or two copies of the *FTO* rs9939609 risk allele. *FTO* gene polymorphism may counteract the beneficial effects of vitamin D in preventing BC. Few studies were performed on the association of *FTO* genotype and vitamin D. In line with the present study, Lourenço et al. reported that effects of *FTO* genotype on child weight gain are more pronounced among children with insufficient serum vitamin D levels.^30^ Another study identified that pre‐surgery vitamin D levels influence the size of genotype effects of *FTO* rs9939609 on RYGB surgery‐induced weight loss in obese patients.^31^ Furthermore, Mehrdad et al. found that intake of vitamin D from sunlight and its nutritional sources and might be solutions for obesity in cases with *FTO* rs9939609 polymorphism.^32^ Interestingly, a recent study identified that the effect of FTO rs9939609 A risk allele on eating behaviour and mental health may be limited to people with insufficient vitamin D intake.^10^ However, another study on the interactions between *FTO* gene polymorphism, depression and serum vitamin D level reported that A‐allele of *FTO* rs9939609 polymorphism might be associated with depression independent of serum vitamin D level.^32^

Based on the available literature, we hypothesized that the A‐allele of *FTO* rs9939609 have an association with vitamin D levels in BC patients. However, in the present study, the patients carrying one or two copies of the *FTO* rs9939609 risk allele had more than 40 ng/dl serum 25 (OH) vitamin D. Vitamin D was reported to be associated with proper insulin secretion and activity,^16^ and lower circulating 25(OH)D has been related to insulin resistance. It is possible that part of the positive effects of vitamin D in preventing disease is due to its role in insulin secretion and function. On the other hand, *FTO* variation was associated with increased reduced peripheral insulin sensitivity.^16^ An underlying reason behind high serum level of vitamin D in the BC patients with the *FTO* risk allele can be that the *FTO* gene polymorphism may counteract the beneficial effects of vitamin D in preventing BC.

This study suggested an intricate relationship between the *FTO* rs9939609 risk allele and serum 25 (OH) vitamin D level in Iranian BC patients. Future studies with a larger sample are needed to confirm these results. The underlying mechanisms of the interactions of the *FTO* gene and vitamin D should be investigated in BC. An investigation targeting the cause‐effect relationship of the risk allele with the molecular signalling pathways such as the oestrogen signalling pathways may help to understand the cause‐effect relationship. In addition, the role of various FTO polymorphisms in vitamin D levels and other biomarkers of BC should be investigated.

### 4.1 Conclusions

In this study, a high level of circulating vitamin D was associated with the *FTO* gene risk allele (A) in BC patients. It may be likely that the *FTO* gene polymorphisms may neutralize the beneficial effects of vitamin D in BC prevention. Further studies are needed to confirm these results and to identify. Further studies on the interaction of *FTO* genes and nutritional factors in BC patients can help to better understand the underlying mechanisms of the effects of *FTO* genotype on the role of vitamin D in BC prevention and to identify the genetic factors predisposing to BC and their effect on the association between vitamin D and BC.

## CONFLICT OF INTEREST

The authors declare that they have no competing interests.

## AUTHOR CONTRIBUTIONS

**Maryam Gholamalizadeh:** Formal analysis (equal). **Zohreh Mokhtari:** Investigation (equal); Supervision (equal). **Saeid doaei:** Investigation (equal); Methodology (equal); Validation (equal). **Vahideh Jalili:** Data curation (equal). **Sayed Hossein Davoodi:** Funding acquisition (equal). **Mona Jonoush:** Software (equal); Validation (equal). **Mohammad Esmail Akbari:** Software (equal); Writing‐original draft (equal). **Azadeh Hajipour:** Investigation (equal); Visualization (equal). **Bojlul Bahar:** Validation (equal); Writing‐original draft (equal). **Ghasem Azizi Tabesh:** Investigation (equal); Visualization (equal). **Saeed Omidi:** Validation (equal); Writing‐original draft (equal). **Seyed Alireza Mosavi Jarrahi:** Formal analysis (equal); Investigation (equal).

## Data Availability

The data sets used and/or analysed during the current study are available from the corresponding author on reasonable request.
